# Open- and closed-source LLMs in medical and engineering education

**DOI:** 10.3389/fmed.2025.1751813

**Published:** 2026-01-13

**Authors:** Liping Sun, Ya Li, Hongxing Kan, Jianhua Shu, Huanqing Xu, Chengle Li, Guokun Shi, Ziyang Wang, Xueqi Wang, Li Jin

**Affiliations:** School of Medical Informatics Engineering, Anhui University of Chinese Medicine, Hefei, Anhui, China

**Keywords:** AI, DeepSeek, large language models, medical and engineering education, prompt engineering

## Abstract

The rapid development of large language models (LLMs), such as the close-source GPT-4, have revolutionized education in assisting students learning. However, open-source LLMs, which have many advantages of accessibility, customization, and transparency, remains under-utilized in both medical and engineering education. The work systematically evaluates the performance of open-source LLMs (DeepSeek, GLM-4, Kimi) and close-source GPT-4 in assisting medical and engineering students learning through diverse question types. We found that DeepSeek outperformed other models for all question types, achieving the highest accuracy rates. To further improve LLM-generated responses, prompt engineering strategies, such as role-playing, generated knowledge prompting, chain-of-thought prompting, few-shot prompting, and output style, were introduced. Post-training evaluations showed significant improvements in model accuracy, with DeepSeek exceeding 95% accuracy for all question types. Among them, Short-answer questions achieved the best response, with the accuracy rate reach up to 97% across four LLMs, indicating the important role of prompt engineering in problem-solving task. The findings highlight the potential of open-source models in supporting medical and engineering education, bridging a critical gap in open-source LLM evaluation and advocating for their wider integration into academic settings.

## 1 Introduction

In recent years, large language models (LLMs), such as Generative Pre-trained Transformer (GPT-4), demonstrated superior capabilities in natural language processing (1, 2). LLMs are trained on a great deal of text data, which could generate human-like text, answer questions, and complete other language-related tasks with the high accuracy. LLMs have been widely applied in numerous areas, such as finance (3), law (4), medicine (5), scientific research (6–8) and education (9–11). In education, LLMs serve as powerful assistant for both teachers and students. For teachers, LLMs support lesson planning, personalized content creation, differentiation and personalized instruction, assessment, and professional development. For instance, ChatGPT was utilized as an instructor’s assistant in generating and scoring examinations (12). A meta-analysis revealed that GPT-4 achieved an overall accuracy rate of 81% across multiple national medical licensing exams, significantly outperforming GPT-3.5’s 58% accuracy (13). Meanwhile, the MedExamLLM platform integrated performance data from 16 LLMs across 198 medical exams, which further confirmed that GPT-4 passed 50% of medical exams (14). These studies provide crucial evidence for the potential application of LLMs in medical education while underscoring the necessity for systematic evaluation.

For students, LLMs could assist in reading, writing, math, science, and language skills, as well as offering personalized practice materials, summaries, and explanations, which largely improved the learning experiences of students (15–18). ChatGPT was applied for writing assistance and evaluation assignments in lower- and upper-division students across the chemistry curriculum (19). A ChatGPT-Assisted Special Topics Writing Assignment in Biochemistry was implemented and evaluated (20). Recently, lots of research extensively tested the potential of ChatGPT in the educational context by evaluating answers given by ChatGPT. Lukas et.al. performed a systematic empirical assessment of ChatGPT’s abilities to answer questions across the natural science and engineering domains (21). Watts et al. compared student and three versions of generative AI chatbots (ChatGPT-3.5, ChatGPT-4, and Bard) responses to two different organic chemistry writing-to-learn assignments. They found the differences between chatbot and students responses (22).

All the above-mentioned efforts were focused on closed-source ChatGPT LLMs. While ChatGPT have obtained widespread attention, in recent years, the domestic open-source LLMs also made significant progress in developing competitive alternatives (23–26). On October 9, 2023, Moonshot AI Company Kimi released the first version of Kimi models, which was the world’s first LLM that supports the input of 200,000 Chinese characters (27). Then, Zhipu AI released open-source GLM-4 on January 16, 2024, which demonstrates powerful visual capabilities comparable to OpenAI GPT-4 (28). On January 27, 2025, DeepSeek, a new open-source LLM, was released and rapidly gained worldwide attention, which taking over the user market to become the No.1 downloaded AI app (29, 30). Open-source LLMs offered comparable performances with the ChatGPT model for certain tasks (29). In addition, open-source LLMs exhibit many advantages over closed-source GPT models in terms of accessibility, customization, transparency, and ease of fine-tuning, making them potential candidates for the education (31). Nevertheless, the application of open-source LLMs in education-especially in medicine and engineering-remains largely unexplored. A systematic evaluation of their performance is a necessary precursor to broader adoption.

**FIGURE 1:**
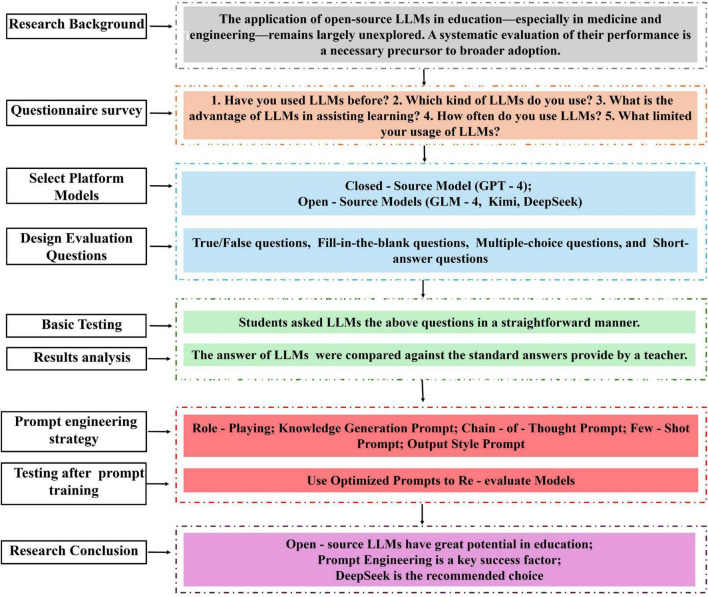
The workflow of this study.

In the work, we assess the performance of open-source LLMs (DeepSeek, GLM-4, Kimi) and the closed-source GPT-4 in assisting medical and engineering students. The workflow of this study is illustrated in Figure 1. A questionnaire survey was conducted to explore how and why students use LLMs. Based on the survey results, we conducted an objective performance evaluation of LLMs using domain-specific academic questions. DeepSeek outperformed the other LLMs for all question types. To further improve LLMs-generated answer, prompt engineering strategy were designed, which contain Role-Playing, Generated Knowledge Prompting, Chain-of-Thought Prompting, Few-Shot Prompting, and Output Style Prompting. After training on prompt engineering strategy, students re-evaluated the performance of LLMs using optimized prompts. Prompt engineering significantly improved the accuracy and reliability of LLMs, especially for DeepSeek, the accuracy rates of which exceeded 95% for all question types after prompt engineering. For four types of questions, Short-answer questions gained the best response with the accuracy rate reaching up to 97%, indicating the crucial role of prompt engineering in problem-solving task. This study underscores the potential of open-source LLMs, especially DeepSeek, in supporting student learning in both medical and engineering education, thereby addressing a critical gap in the literature and encouraging further integration of open-source AI tools in academia.

## 2 Materials and methods

### 2.1 Participants and survey procedure

To assess LLMs usage in medical and engineering students, 400 sophomores with the age range at 19–20 years were involved in the survey. Students were eligible to participate if they were: (1) second-year undergraduates, (2) enrolled in one of the specified medical or engineering majors. A 57% of students majored in medicine (such as Medical Imaging and Pharmaceutical Engineering) and 43% of students majored in engineering (such as Biomedical Engineering and Medical Information Engineering). The gender distribution participants was 52% for male and 48% for female. We conducted the questionnaire survey online using the Chaoxing mobile learning platform. All data were collected from September to December, 2024. All participants agreed to the use of the data for scientific purposes. The survey contained the follow five multiple-choice questions: 1. Have you used LLMs before? 2. Which kind of LLMs do you use? 3. What is the advantage of LLMs in assisting learning? 4. How often do you use LLMs? 5. What limited your usage of LLMs? The survey was collected and analyzed to evaluate the usage of LLMs. The survey results were used to select LLMs for the subsequently performance evaluations.

### 2.2 Model selection

Based on the prominence in the current market and the reported usage by students in our preliminary survey, four LLMs (DeepSeek, GPT-4, GLM-4 and Kimi) were selected to investigate LLMs’ performance in in assisting medical and engineering students’ study. The specific model versions accessed were: GPT-4-1106 (via OpenAI API); DeepSeek-V3 (accessed via the official web interface); GLM-4-Plus (accessed via the official web interface); Kimi Chat (accessed via the official web interface). All evaluations used the default settings and parameters available to a standard user at the time. No custom configurations were modified.

### 2.3 Prompt engineering strategies

To optimize LLM responses for the educational assessment context, we designed and applied a structured prompt engineering framework. (1) Role-Playing: Assign LLMs a professional role to guide their responses. LLMs could generate outputs according to the area of expertise related to the role you assign it; (2) Generated Knowledge Prompting: It requires LLMs to generate relevant background knowledge before addressing the primary question, which ensures the response is well-informed and contextually appropriate; (3) Chain-of-Thought Prompting: It requires LLMs to show its reason, which is very useful for problem-solving and complex question where intermediate steps are crucial; (4) Few-Shot Prompting: It means to provide a few examples to guide the LLM’s response; (5) Output Style Prompting: It ensures that the response is organized and presented in a clear manner.

### 2.4 Answers collection

The Electrical and Electronic Engineering course were selected for this study, because it covers foundational topics such as circuit analysis, signal processing, and biomedical instrumentation, which is highly relevant to both engineering and modern medical education. In medical fields, such as Medical Imaging and Biomedical Engineering, understanding electronic principles is essential for operating diagnostic equipment, interpreting imaging data, and developing medical devices. Thus, using questions from this course allows us to assess LLMs’ ability to handle interdisciplinary content that bridges engineering and medical domains, ensuring the evaluation reflects real-world educational scenarios encountered by students in both disciplines. Questions and the corresponding standard answers was provided by a teacher of Electrical and Electronic Engineering course, which were presented in Chinese. The questions included 32 True/False questions, 11 Fill-in-the-blank questions, 23 Multiple-choice questions, and 28 Short-answer questions. Students asked LLMs the above questions in Chinese by a straightforward manner. The responses provided by LLMs were recorded. After the straightforward interaction, students were systematically trained on prompt engineering strategy. The prompt engineering strategy contained Role-Playing, Generated Knowledge Prompting, Chain-of-Thought Prompting, Few-Shot Prompting, and Output Style Prompting. Then, students posed the same questions to LLMs by the prompt engineering strategy and recorded the answers.

### 2.5 Data analysis

The answer of LLMs in each round were compared against the standard answers provide by a teacher. For True/False, Multiple-choice, and Fill-in-the-blank questions, the answer of LLMs were marked as correct when they matched the standard answer. For fill-in-the-blank questions, when the core technical term or value was identical, minor grammatical variations were accepted. For Short-answer Questions, each standard answer was decomposed into a pre-defined set of essential key points by a teacher. A teacher scored each LLM response by assessing the presence and accuracy of each key point. A response was classified as correct when the accuracy rate reached up to 0.8. The accuracy rate of each question type was calculated by dividing the number of correct answers by the total numbers of questions. The accuracy rates of LLMs were compared between the straightforward manner and the prompt engineering strategy. All participants agreed to the use of the data for scientific purposes.

## 3 Results

### 3.1 Analysis of questionnaire

**FIGURE 2:**
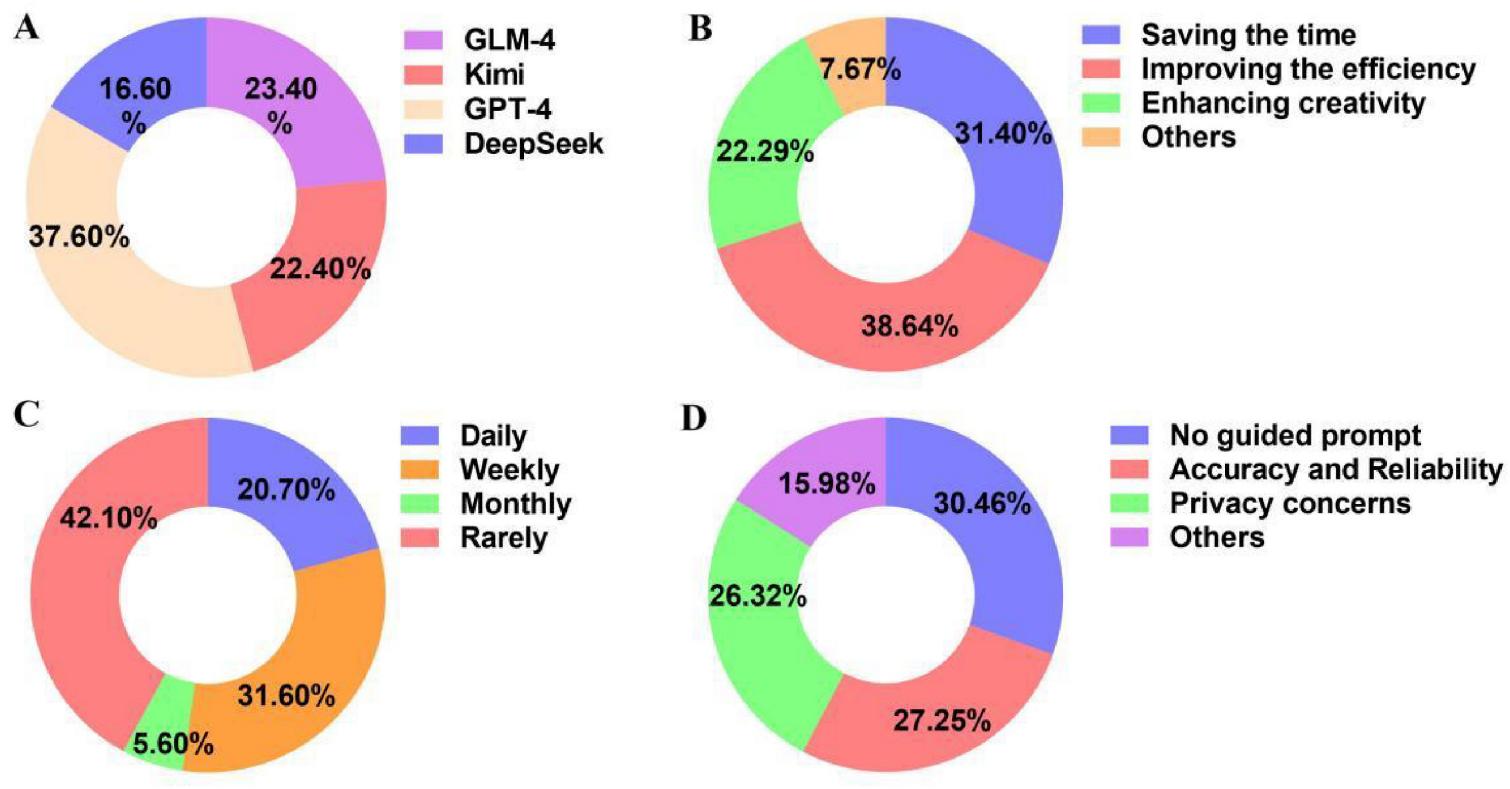
Students’ questionnaire analysis. **(A)** Types of LLMs used by students. **(B)** Advantages of LLMs in assisting learning. **(C)** The frequency of LLMs used by students. **(D)** Limiting factors of LLMs used by students.

To evaluate the use of LLMs in their learning process, 400 students were participated in the questionnaire. The results demonstrated that 91.2% of students used LLMs for study, indicating that LLMs have becomes an important academic toolkit. With the advent of GPT-4, numerous LLMs have been developed. Because of the transparency and accessibility, open-source models, such as Kimi, GLM-4, DeepSeek, demonstrated many advantages. 23.4, 22.4, and 16.6% of students used GLM-4, Kimi or DeepSeek, respectively (Figure 2A). 31.4% of students agreed that LLMs significantly saved their time. 38.64% of students improved their learning efficiency by applying LLMs in their study (Figure 2B). However, only 20.7% of students used LLMs daily, 42.1% of students just occasionally used LLMs (Figure 2C). The main reasons that restrict the wide application of LLMs are their inability to effectively use prompts (30.46%) and concerns about the accuracy of LLMs’ responses (27.25%) (Figure 2D). Therefore, it is necessary to improve students’ ability to use prompt, which could further improve the accuracy of LLMs’ response.

### 3.2 Evaluation of open-source and closed-source LLMs in assisting medical and engineering students’ study without prompt engineering

**FIGURE 3:**
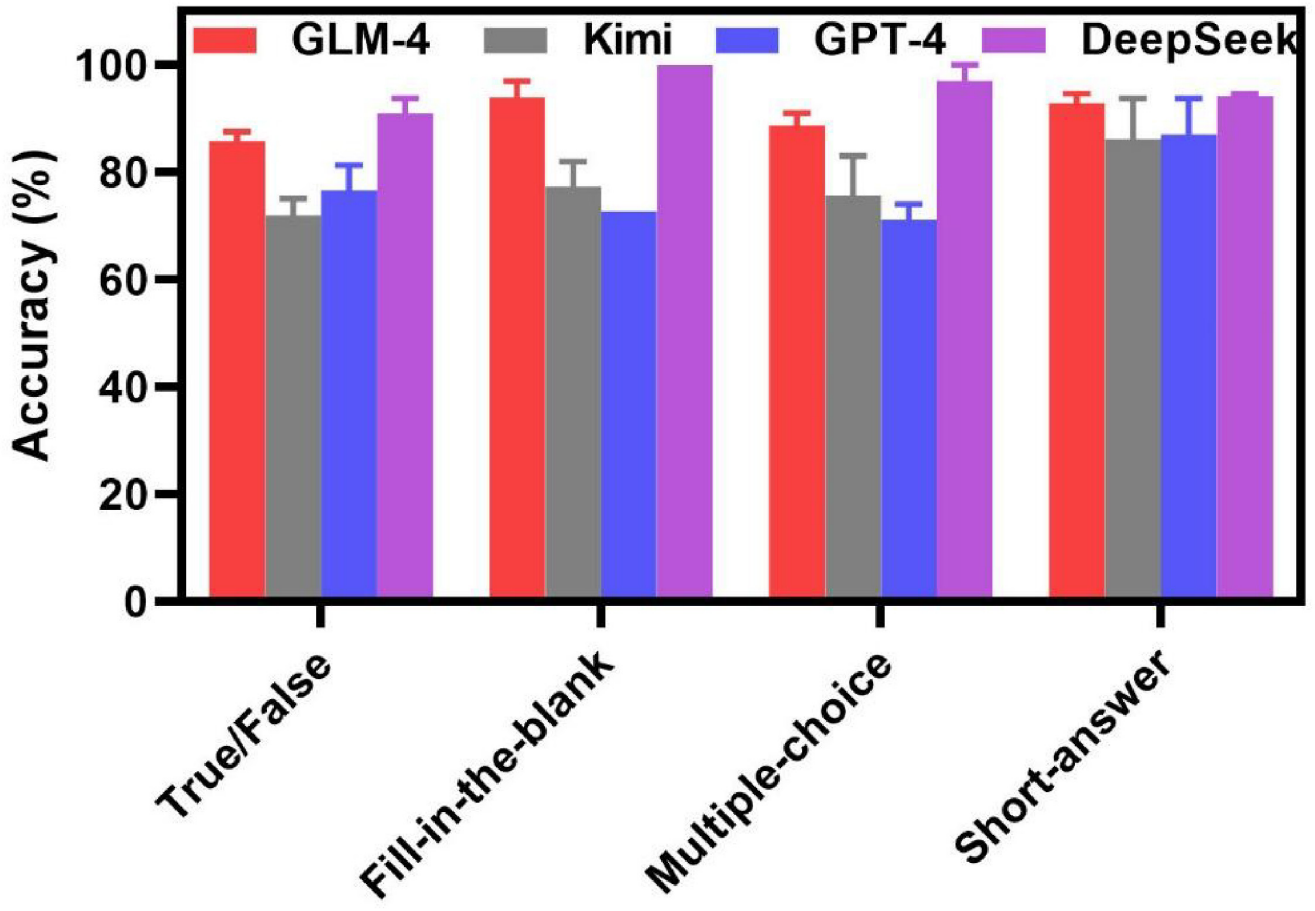
The accuracy of open-source and closed-source LLMs in assisting students’ study without prompt engineering.

In the work, we evaluated the open-source and closed-source LLMs for their application in assisting medical and engineering students’ study. A Comparison of open-source and closed-source LLMs for Educational Use was summarized in Table 1. A set of questions from Electrical and Electronic Engineering course were devised by the teacher to assess the performance of three open-source LLMs (DeepSeek, GLM-4 and Kimi) and a closed-source LLM (GPT-4). The questions contain four types, which were True/False, Fill-in-the-blank, Multiple-choice, and Short-answer questions. Students asked questions in a straightforward manner. As can been seen in Figure 3, the accuracy rates of DeepSeek for True/False, Fill-in-the-Blank, Multiple-Choice, and Short-answer questions were 90.775, 100, 97, 94.195%, respectively. In contrast, the accuracy rates of GLM-4 for True/False, Fill-in-the-Blank, Multiple-Choice, and Short-answer questions were 85.75, 93.9, 88.68, and 92.855%, respectively. Kimi’s accuracy rates for the above question types were 71.875, 77.35, 75.59, and 86.16%, respectively. For GPT-4, the accuracy rates for True/False, Fill-in-the-Blank, Multiple-Choice and Short-answer were 76.625, 72.735, 71.09, and 87.055%, respectively. Our findings reveal that DeepSeek outperformed the other three LLMs for all question types, which hold significant potential in medical and engineering study. Meanwhile, low accuracy of the other LLMs in the field of medical and engineering education increased our attention. It is very important to train students on the reflective and informed use of LLMs.

**TABLE 1:** A Comparison of open-source and closed-source LLMs for educational use.

Feature	Open-source LLMs	Closed-source LLMs
Cost	No cost for base access; potential for self-hosting.	Recurring subscription or per-token API fees; can be cost-prohibitive at scale.
Deployment and control	Can be deployed on-premises or private servers; offers greater data privacy and control.	Cloud-based API access only; reliance on provider’s infrastructure and policies.
Customizability and adaptability	High; weights and architectures can be fine-tuned for specific domains	Limited; primarily accessible via prompting and provided APIs; no model weight modification.

### 3.3 Observational outcomes of prompt engineering

**FIGURE 4:**
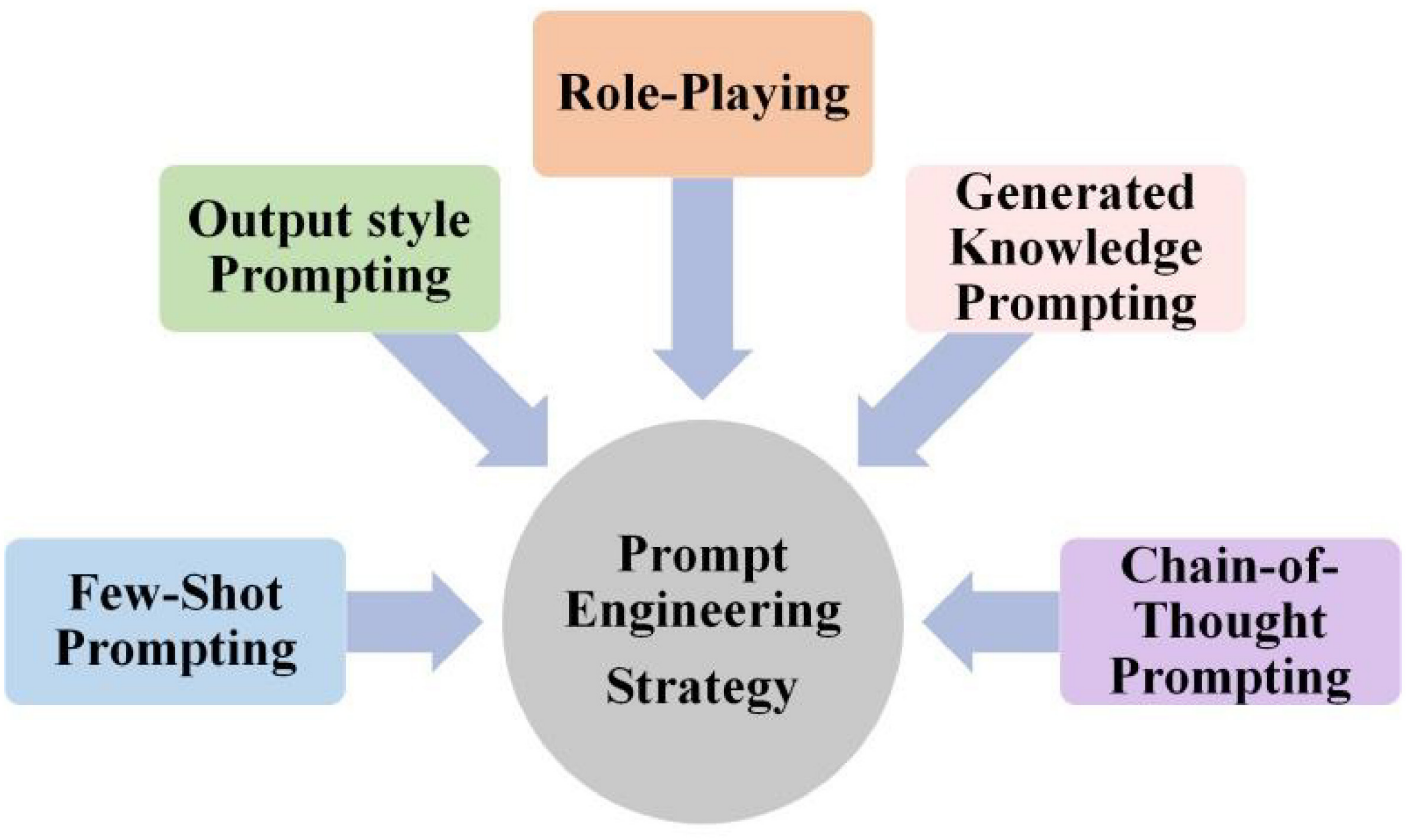
Prompt engineering strategy.

**FIGURE 5:**
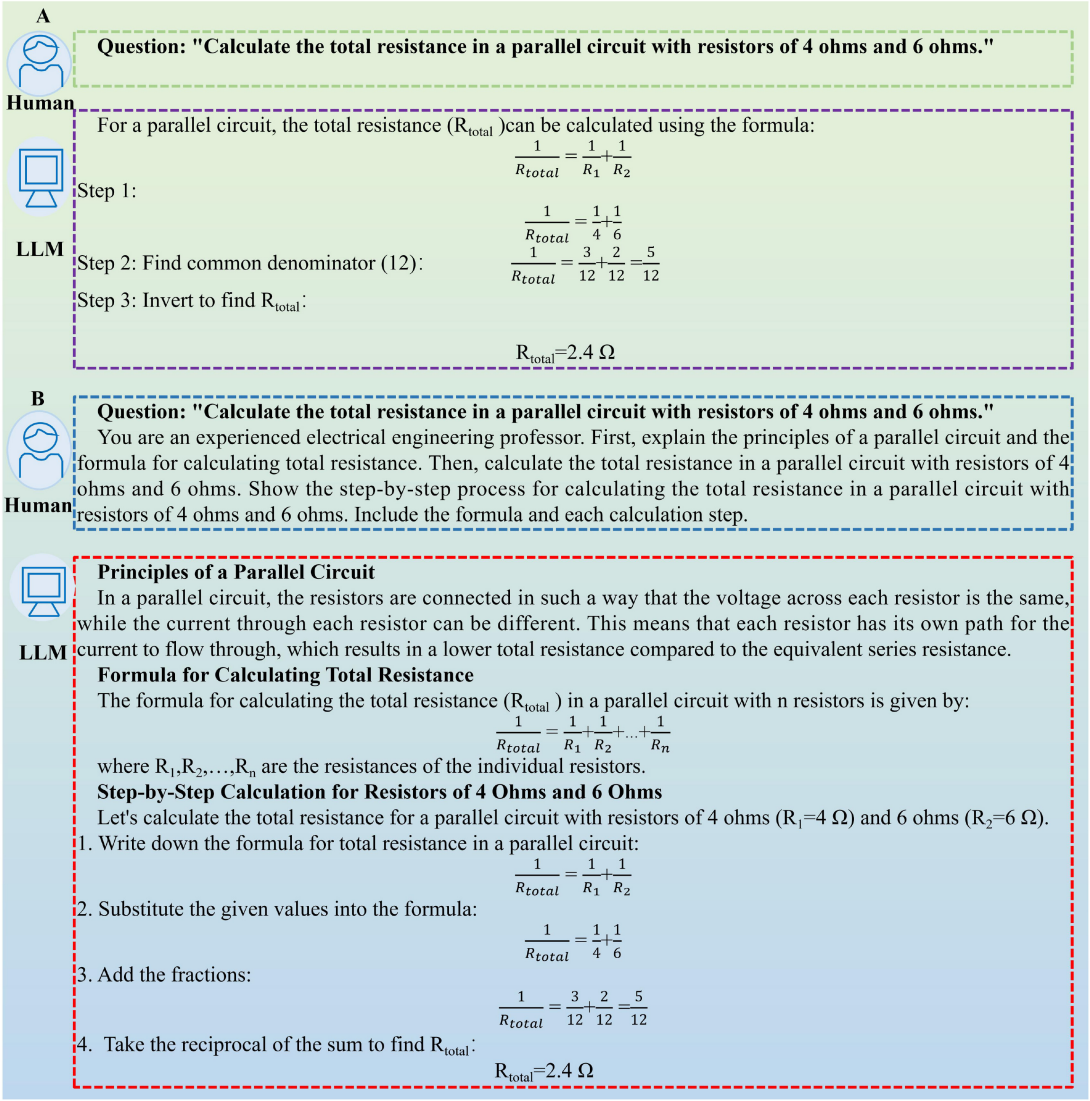
Example of using DeepSeek for generating response. **(A)** No prompt. **(B)** With prompt engineering strategy.

To improve the quality and usability of LLM-generated answers, we optimized the prompt engineering, which contained Role-Playing, Generated Knowledge, Chain-of-Thought, Few-Shot and Output Style (Figure 4). Responses from the prompt engineering group evolved significantly. They adopted a more authoritative, pedagogical voice. Logical reasoning was presented in clear, traceable steps. These steps were inherently verifiable. The entire structure resembled an expert’s explanation for a learner. Figure 5 illustrates the response of DeepSeek for the question: “Calculate the total resistance in a parallel circuit with resistors of 4 and 6 ohms.” For no prompt group, although DeepSeek’s response was correct, the train of thought was typically terse and lacked pedagogical framing. For prompt engineering group, we assigned the role as an experienced electrical engineering professor. We crafted a prompt with two distinct requirements. DeepSeek had to detail the workings of a parallel circuit. It also needed to provide the total resistance equation. This method ensured the response was grounded in relevant background knowledge. By providing a step-by-step guide on how to calculate the total resistance in a parallel circuit with resistors of 4 and 6 ohms, DeepSeek could generate responses that aligned with educational needs. This structured output is more valuable for educational scaffolding and self-study purposes.

### 3.4 Comparison of LLMs performance by prompt engineering strategy

**FIGURE 6:**
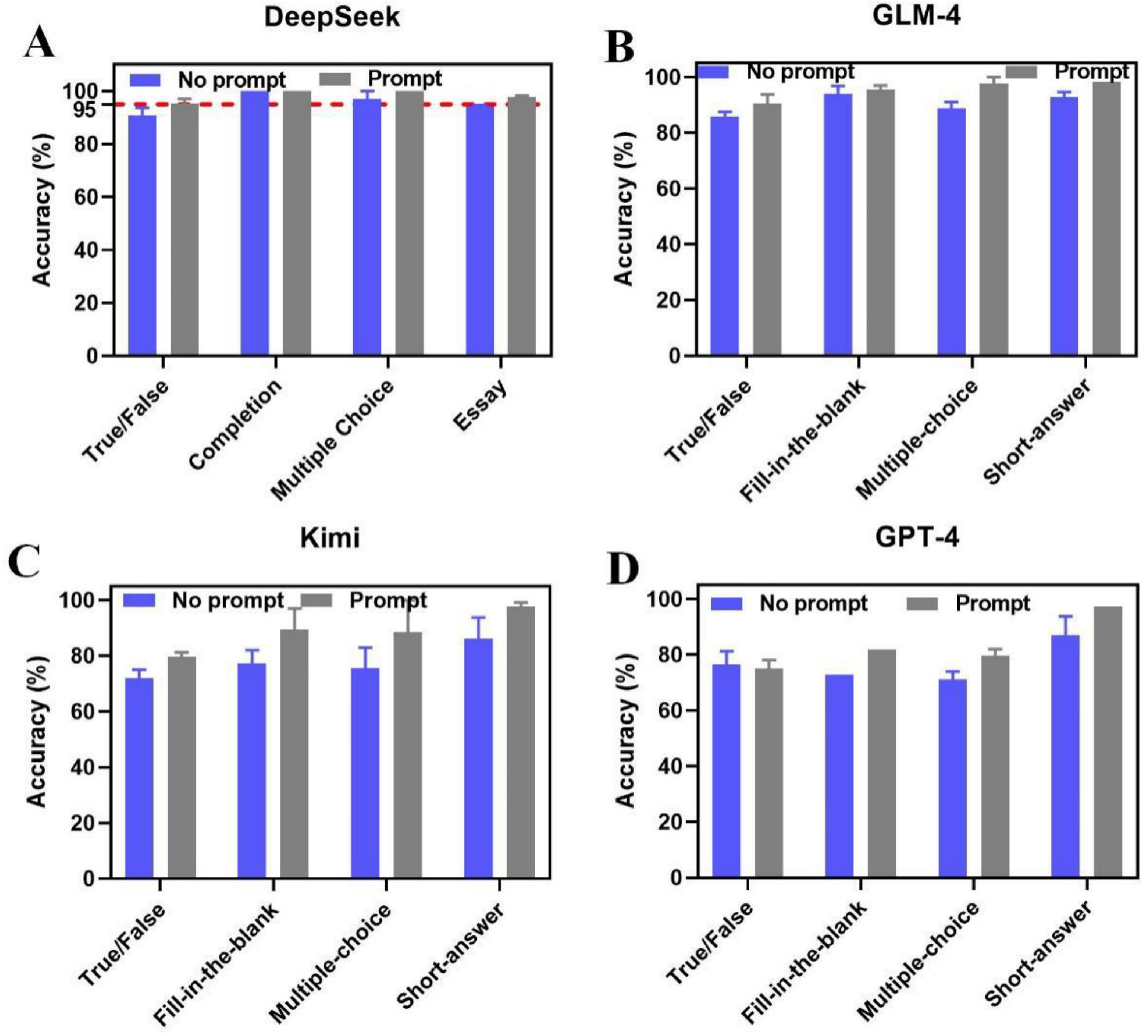
The accuracy of **(A)** DeepSeek, **(B)** GLM-4, **(C)** Kimi, **(D)** GPT-4 in assisting students’ study with or without prompt engineering.

After training on prompt engineering strategy, students were required to re-evaluate the performance of LLMs using optimized prompts engineering strategy. As can be seen from Figure 6, the accuracy rates of DeepSeek reached up to 95.375, 100, 100, and 97.765% for True/False, Fill-in-the-Blank, Multiple-Choice, and Short-answer questions, respectively. For GLM-4, the accuracy rates were 90.625% for True/False questions, 95.485% for Fill-in-the-Blank questions, 97.725% for Multiple-Choice questions, and 98.21% for Short-answer questions. Kimi’s accuracy rates for the four question types were 79.6875, 89.485, 88.5, and 97.765%, respectively. However, GPT-4 obtained accuracy rates of 75% for True/False questions, 81.82% for Fill-in-the-Blank questions, 79.635% for Multiple-Choice questions, and 97.32% for Short-answer questions. Compared with the accuracy rates without prompting, prompt engineering significantly improved the accuracy and reliability of LLMs, especially for DeepSeek, the accuracy rates of which exceeded 95% across all question types after prompt engineering. For four question types, Short-answer questions achieved the best response with the accuracy rate reaching up to 97%, indicating the crucial role of prompt engineering in problem-solving task. In the future, more sophisticated prompt specific for specific field could be developed. What’s more, it is also necessary to longitudinally investigate the long-term effect of LLMs on medical and engineering students’ study.

## 4 Discussion

Large Language Models (LLMs) are transforming education. Student interaction with academic courses is fundamentally altered. Our study systematically investigated four LLMs: GPT-4, GLM-4, Kimi, and DeepSeek. We tested their ability to assist in medical and engineering studies. Although LLMs have been widely applied in assisting students’ learning, there is still much to be understood. Ineffective prompt engineering is a key concern. Accuracy issues also persist. Without prompt engineering, open-source models DeepSeek demonstrated remarkable performance in answering domain-specific questions compared to that of closed-source GPT-4. Structured prompt engineering proved highly effective. Prompt engineering strategy included Role-Playing, Generated Knowledge, CoT, and Output Style. These methods significantly boosted response accuracy. They also enhanced the pedagogical quality for all models.

Nevertheless, there still exist some methodological limitations. The participant pool was not diverse. All 400 students came from a single university, Anhui University of Chinese Medicine. Their familiarity with LLMs is specific to a Chinese educational context. This may limit the generalizability of our findings. Adoption rates and performance might be overestimated for global settings. Students may have overstated their LLM usage frequency. They might have exaggerated the advantages. This tendency likely skewed the questionnaire survey results. Our evaluation was also narrow. It focused on a single engineering course. The results may not apply to other academic disciplines. Finally, while prompt engineering strategy was systematically applied, the subjective scoring of Short-answer questions were based on key points, which may lead to evaluator bias. Future research should address these by expanding to larger, multi-institutional samples. Objective metrics, such as learning outcome tests, should be incorporated. Longitudinal studies are needed to validate long-term benefits on student performance.

## 5 Conclusion

In this study, medical and engineering students’ use of LLMs for study was investigated. The survey confirmed the popular of LLMs in assisting students’ study. By comparing the performance of open-source LLMs (DeepSeek, GLM-4, Kimi) and closed-source LLMs (GPT-4) in answering questions, we found that open-source DeepSeek demonstrated excellent response for all question types. Students initially lacked prompting skills. We provided training in prompt engineering, which included Role-Playing, Generated Knowledge, CoT, Few-Shot, and Output Style prompting. Prompt engineering strategies significantly improved LLM accuracy and reliability. The effect was most pronounced for DeepSeek. Its accuracy rate exceeded 95% on all question types. Across all four LLMs, short-answer questions received the best responses. Their accuracy rate reached 97%. This result highlights the importance of prompt engineering for problem-solving tasks. In conclusion, our work reveals two key insights. Open-source DeepSeek has significant potential. Prompt engineering plays a crucial role in assisting students. Future work should address this trend. Advanced prompts tailored to specific majors will be necessary. This will ensure LLM accuracy in educational settings.

## Data Availability

The datasets presented in this study can be found in online repositories. The names of the repository/repositories and accession number(s) can be found in this article/supplementary material.
